# Outcome and complications of percutaneous nephrolithotomy as primary versus secondary procedure for renal calculi

**DOI:** 10.1590/S1677-5538.IBJU.2014.0619

**Published:** 2016

**Authors:** S. V. Krishna Reddy, Ahammad Basha Shaik

**Affiliations:** 1 Department of Urology, Narayana Medical College, Nellore, Andhra Pradesh, India; 2 Department of Statistics, Sri Venkateswara University, Tirupati, Andhra Pradesh, India

**Keywords:** Kidney Calculi, Nephrostomy, Percutaneous, complications [Subheading]

## Abstract

**Purpose:**

To compare the efficacy of percutaneous nephrolithotomy (PCNL) as a primary procedure of patients following previous open surgery or post percutaneous nephrolithotomy (PCNL) for renal calculi.

**Materials and Methods:**

The medical records of 367 patients who underwent PCNL by a single surgeon from January 2008 to December 2013 were reviewed retrospectively. All patients were divided into 3 Groups. Group-1 (n=232) included patients with no history of ipsilateral open stone surgery. Group 2 (n=86) patients had undergone one or more open stone surgeries before PCNL, patients with failed or recurrence following PCNL were placed in Group-3 (n=49). The demographic data, operation duration, stone free rate (SFR), number of attempts to access the collecting system and intra operative and postoperative complications between the three Groups were compared.

**Results:**

There was no difference in sex, Body Mass Index (BMI), stone burden and laterality among the three Groups. Operation time was significantly less in first Group, while there was a statistically significant difference in operation duration between second and third Groups (p<0.05). The number of attempts to enter the collecting system was lower in the first Group in comparison to other two Groups (p<0.5). There was no significant differences among three groups in stone free rate. Intra operative and postoperative complications were slightly more frequent in Groups 2 and 3. Mortality occurred in 1 patient with colon perforation in Group-2.

**Conclusion:**

Our study demonstrated that PCNL can be performed in patients even as secondary procedure without further complications.

## Introduction

Urolithiasis has long plagued human civilization. Management of patients suffering from urinary tract calculi is considered to be a health care problem because of its prevalence and recurrence. Renal stone treatment has significantly evolved from open surgery to minimal invasive surgical procedures. Since the first report of the removal of renal stones via nephrostomy by Rupel and Brown in 1941 (1), there have been significant improvements in techniques, instruments, and experience. Fernastom and Johansson first reported percutaneous nephrolithotomy (PCNL) in 1976 (2). Alken et al. introduced the renal endoscope and ultrasonic lithotripsy to further development of the technique. Although extracorporeal shock wave lithotripsy (ESWL) and flexible ureteroscopic stone removal are widely used modalities for renal stones, PCNL is still needed for selected cases according to the size, position, shape, and composition of the stones (3). Recently European Association has considered PCNL as first option for large, multiple or inferior calyx stones (4). Open stone surgery has been replaced by PCNL because of its cost effectiveness, lower morbidity, shorter operative time and lower postoperative complications (5, 6). Some patients with history of open stone surgery need PCNL because of renal stone recurrences (7, 8). Stone recurrence rate is up to 50% within 5–7 years (9). PCNL or open stone surgery causes scar tissue and anatomical modifications in kidney that may affect later PCNL. Some studies have reported that open stone surgery can increase PCNL failure rate (10) while others show that previous open stone surgery does not affect PCNL outcome (11, 12). PCNL is recommended for cases with stones larger than 20mm^2^, cases with struvite or cystine stones, cases in which stone removal failed with ESWL, or cases accompanied by anatomical malformation (5, 13). However, PCNL does carry a risk of significant morbidity, with contemporary series describing a complication rate of 20.5% (14).

The aim of our study was to compare PCNL efficiency and complications in patients with and without history of open renal stone surgery and in patients following failure or recurrence following PCNL procedure.

## Materials and Methods

The medical records of 367 patients who underwent PCNL by a single surgeon from January 2008 to December 2013 were reviewed retrospectively. Our study was approved by our institutional ethics committee. Written informed consent was taken from all patients for photographing, recording and also its use for scientific and medical education purposes. All patients were divided into 3 Groups. Table-1 compares the demographic profile of all the three groups. Group-1 included primary patients with no history of ipsilateral open stone surgery or PCNL procedure (n=232). Group-2 included patients who had undergone one or more open stone surgery before PCNL (n=86). Group-3 included patients with failed PCNL for renal calculi or recurrence following previous PCNL procedure (n=44). The indications for PCNL included a stone burden of greater than 20mm^2^ in length or failure of 2 to 3 attempts of ESWL treatment with stone burden of ≥10.5mm^2^. Patients with Body Mass Index (BMI) ≥30, patients with abnormal renal anatomy such as ectopic or horse shoe kidneys and a stone burden of more than 700mm^2^ were excluded from the study. The stone burden was measured as the product of the two dimensions on plain radiographs. All patients were evaluated with renal function test, blood counts, coagulation profile, urine routine, urine culture sensitivity and ultrasonography. An intravenous urography (IVU) was carried out in all to assess function and planning of the puncture. Urinary tract infections detected preoperatively were treated according to antibiotic sensitivity. Computed tomography (CT) scan was performed in patients with history of open surgery. Patients with retro-renal colon in CT scan were candidate for open stone surgery. After general anesthesia, a 5 or 6 French (F) ureteral catheter was inserted and fixed to a Foley catheter. Patients were then turned into a prone position with special care for the pressure points. Trans-papillary puncture was made preferably away from the previous incision site if any, using a three part needle (Angiomed 1.3mm (17.5G)) under fluoroscopy control after retrograde opacification of the pelvi-caliceal system via ureteral catheter. An angle tip Terumo® wire was then positioned in the upper ureter. The tract was then dilated initially using serial Teflon dilators up to 10Fr, followed by placement of Alken's rod. The subsequent tract dilation was performed by serial metallic or Teflon dilators. After Amplatz sheath insertion, nephroscopy was performed and stones were fragmented by a pneumatic lithotripter and removed. Normal saline was used for continuous irrigation. If there was more than 20mm^2^ residual stone that could not be accessed from the first tract, a second access was established. The fragmented calculi were removed using forceps or suction. On the Table, complete clearance was ensured by fluoroscopy and direct nephroscopy. An adequate size nephrostomy was placed at the end of the procedure. Nephrostomy was removed on the second postoperative day after performing X-ray KUB and abdominal ultrasonography or CT scan (for radiolucent stones) to determine the residual stones. The nephrostomy tract site was closed with sterile dressing. Patient was then discharged with the instructions to remove the dressing after 72 hours and follow-up after one week if asymptomatic. Patients' age, sex, Body Mass Index (BMI), stone burden, laterality, operative duration, length of hospital stay, number of attempts before successful entry into collecting system, stone free rate (SFR) and intra-operative and postoperative complications rate were compared between three groups.

**Table 1:** Demographic profile of patients in all the three groups.

Parameters		Group 1 (n=232)	Group 2 (n=86)	Group-3 (n=49)	P Value
Mean Age (years)		25.54±5.55	45.67±13.21	42.68±9.92	<0.0001*
**Sex**					
	Males	151 (65.1%)	52 (60.5%)	31 (63.3%)	0.74
	Females	81 (34.9%)	34 (39.5%)	18 (36.7%)	
BMI (kg/m^2^)		22.81±5.43	22.40±5.81	22.48±4.73	0.808
Stone size (mm^2^)		22.84±1.07	23.04±0.99	22.97±0.96	0.28
**Stone Side**					
	Right	0.89±0.54	1.04±0.44	0.95±0.44	0.055
	Left	1.02±0.62	1.2±0.52	1.1±0.48	0.051
**Location of Stone**					
Calyceal		69 (29.7%)	24 (27.9%)	15 (30.6%)	0.96
Pyelocalyceal		94 (40.5%)	39 (45.3%)	20 (40.8%)	
Pelvic		69 (29.7%)	23 (26.77%)	14 (28.6%)	
**Number of Stones**					
	Single	177 (76.3%)	67 (77.9%)	36 (73.5%)	0.84
	Multiple	55 (23.7%)	19 (22.1%)	13 (26.5%)	
**Opacity**					
	Radio opaque	173 (74.6%)	65 (75.6%)	36 (73.5%)	0.96
	Radiolucent	59 (25.4%)	21 (24.4%)	13 (26.5%)	
**Preoperative hydronephrosis**					
	None	59 (25.4%)	21 (24.4%)	11 (22.4%)	0.99
	Mild	57 (24.6%)	22 (25.6%)	14 (28.6%)	
	Moderate	60 (25.9%)	22 (25.6%)	14 (28.6%)	
	Severe	56 (24.1%)	21 (24.4%)	10 (20.4%)	

* **P<0.0001** = Very high significant; **p<0.05** = significant; **p>0.05** = Not significant

### Statistical analysis

Data values were entered into MS-Excel and statistical analysis was done by SAS Version 9.2 (SAS Institute Inc., Cary, NC, USA). For categorical variables, the values were expressed as number and percentages and to test association between the three Groups, the chi-square test was used. For continuous variables, the values were expressed as mean±standard deviation and to test mean difference between the three Groups, the ANOVA test with Post hoc test was used. All p-values less than 0.05 were considered as statistically significant.

## Results

**Figure 1:**
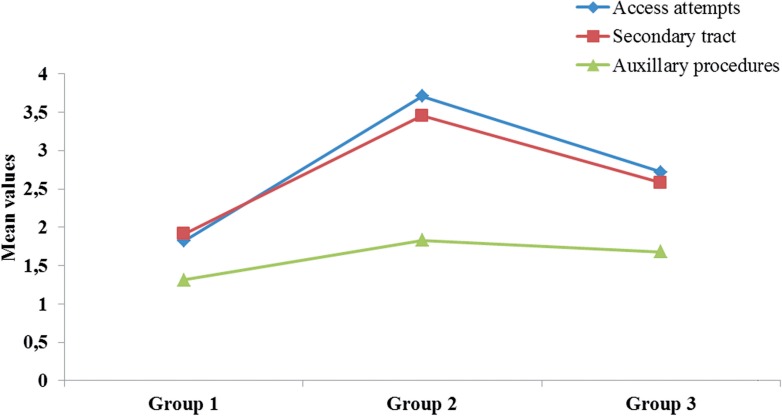
Comparison between three groups of access attempts, secondary tract & auxiliary procedures.

Table-2 shows the overall outcome in all three Groups. All Groups were comparable in terms of Body Mass Index (BMI), stone laterality, number of stones, opacity, stone burden and preoperative hydronephrosis. Patients mean age of Group 2 (45.67±13.21) and Group 3 (42.68±9.98) were significantly higher (P<0.0001) than in Group 1 (25.54±5.55). Mean operative time (in minutes) for Group 1 was shorter (76.24±19.47) as compared with Group 2 (83.67±19.83) and Group 3 (83.17±17.37), which was statistically significant. The majority 90.69% (78/86) of the patients in Group 2 had undergone one surgery in the past, except eight (6.97%) of which had undergone open surgery twice and two (2.33%) three times. The majority 73.5% (36/49) of Group 3 patients had stone recurrence following PCNL which was more than 10.5 to 20mm^2^ and ten (20.4%) patients had failed PCNL after 2 to 3 attempts. The average time from last open surgery or PCNL recurrence to the present percutaneous procedure was 5±2.3 years. The attempts to access the pelvi-calyceal system (PCS) in Group 1 patients (1.82±0.49) was significantly less when compared with Group 2 (3.71±0.56) and Group 3 (2.72±1.12) patients respectively (Figure-1) (p<0.0001). We observed that when the approach in Group 2 was not from the incision site, the dilation was easy compared to access gained from the region of scar tissue. This also reduced the probability of guide wire kinking or access failure. Table-2 also compares the complications within the three Groups. Overall intra-operative bleeding was observed in 11.2% (26/232) in Group 1, 14% (12/86) in Group 2, and 10.2% (5/49) in Group 3 patients requiring blood transfusion. Bleeding responded to conservative measures. Injury to adjoining organ colon on the right side was noted in one patient (1.2%) in Group 2, who developed severe sepsis and died on the fifth postoperative day. Extravasation was defined as the presence of urine in the nephrostomy tract after five days, which was managed conservatively and was not significant in all three Groups. None of our patients developed pseudo aneurysms. However, 6 (2.6%) patients in Group 1, 4 (4.7%) in Group 2 and 2 (4.1%) in Group 3 developed pneumothorax and required chest drain placement. All these patients had upper calyceal second puncture for stone clearance 10.8% in Group 1, 15.1% in Group 2, and 12.2% in Group 3 patients developed postoperative fever and that was attributed to pyelonephritis. These patients were treated conservatively with injectable antibiotics (first-generation cephalosporin's and amino glycoside) until they were afebrile and then switched over to oral therapy (oral quinolone) to complete two weeks of medication. Auxiliary procedures such as second look PCNL and SWL were performed at 1.31±0.56 in Group 1, 1.83±0.59 in Group 2, and 1.68±0.54 in Group 3 patients. Stone free rates post-surgery in all the three Groups were not much different and were not statistically significant.

**Table 2:** Results and Complications in all the three groups;

Parameters	Group-1 (n=232)	Group-2 (n=86)	Group-3 (n=49)	P Value
Mean Operative Time (minutes)	78.24±19.47	83.67±19.83	83.17±17.37	0.043*
Access attempts	1.82±0.49	3.71±0.56	2.72±1.12	<0.0001*
Secondary tract	1.91±0.75	3.45±1.04	2.58±0.53	<0.0001*
Auxiliary procedures	1.31±0.56	1.83±0.59	1.68±0.54	<0.0001*
Average Drop In Hb (gm %)	1.24±0.54	1.41±0.56	1.28±0.63	0.055
Bleeding (Intra-op)	26 (11.2%)	12 (14.0%)	5 (10.2%)	0.824
Blood Transfusion	11 (4.7%)	6 (7.0%)	2 (4.1%)	0.712
Pseudo aneurysm	0	0	0	
Pneumothorax	6 (2.6%)	4 (4.7%)	2 (4.1%)	0.585
Renal pelvic injury	1 (0.4%)	1 (1.2%)	0 (0.00%)	0.641
Damage to adjoining colon	0 (0.0%)	1 (1.2%)	0 (0.0%)	
Post Operative Fever	25 (10.8%)	13 (15.1%)	6 (12.2%)	0.546
Stone Clearance	218 (94.0%)	80 (93.0%)	46 (93.9%)	0.859
Extravasations	5 (2.2%)	3 (3.5 %)	1 (0.02%)	0.791
Hospital stay (days)	3.16±0.90	3.15±0.95	3.14±0.83	0.99

* **P<0.0001** = Very high significant; **p<0.05** = significant; **p>0.05** = Not significant

## Discussion

Surgical management of renal tract stone disease has evolved during the last two decades after the introduction of minimal invasive techniques, like ESWL and PCNL (15). PCNL has become a common procedure performed in patients with renal calculi (16). Since the recurrence rate for renal stones is high, these patients often need re-intervention. Reports have claimed higher failure rates of PCNL in patients with prior open intervention (10, 17). Conversely Shah et al. and Margel et al. in their studies demonstrated that anatomical changes after open stone surgery such as infundibulum stenosis, perinephric fibrosis, bowel displacement and incisional hernia may decrease PCNL success rate and increase its complications (18, 19). Our findings showed that previous open stone surgery or PCNL did not affect subsequent PCNL results and complications. Similar to our findings, a number of studies showed that PCNL can be performed successfully without risk of complications in patients with a history of previous open surgery or PCNL (19–21). The mean operative time in the present study was significantly higher in Groups with single or multiple previous stone surgeries or previous PCNL procedure. Margel et al. and Tugcu et al. have also expressed that operative time was longer in patients with history of previous open nephrolithotomy (19, 20). The factors that may cause prolonged PCNL in patients after open surgery or PCNL are difficulties in tract dilatation in scarred collecting system and perinephric spaces, difficulties in stone fragment removal by grasping forceps and rigid nephroscopy in scarred kidney and cautious fixation of kidney in the retroperitoneum. The rate of auxiliary procedures like second-look PCNL or ESWL was the same in all Groups in our study. Sofikerim et al. and Kurtulus et al. reported the same finding regarding the auxiliary procedures (12, 21). Gupta and colleagues found that relook PCNL is higher in patients with previous open surgery (22). Similar to our results Margel et al. found that access attempts is higher in patients with previous open surgery (19). Puncturing the calyx of interest through the non-operated scar site makes the dilatations easy. Shah et al. preferred a supracostal approach, whereas Basiri et al. suggested a lower calyceal puncture to avoid scar tissue (18, 11). Margel et al. in their study, recommend choosing upper-pole caliceal puncture to avoid the scar tissue coming in the way of the puncture needle (19). But in our study we have selected to access the primary calyx depending upon the stone burden regardless of its relation to scar tissue or ribs. In Sarhad Khan et al. study, febrile urinary tract infection was observed in 8 patients (4%) who were subsequently treated conservatively with parental antibiotics (15). Li MK and Lames S reported symptomatic urinary tract infection in 5.5–9.2% (23, 24). In our study infection requiring antibiotics was seen in 10.7–13.6% in all 3 Groups, which is slightly higher than other studies. We did not have any reports of septicemia or mortality secondary to infection.

PCNL is generally accepted as a safe procedure. Hemorrhage is the most frequent complication of this procedure. Excessive bleeding can occur during needle passage, tract dilatation, or nephrostomy (25–27). Similar to our study acute bleeding requiring transfusion has been reported in 3% to 12% of cases (10, 27, 28). Fortunately, in our study and the Sarhad Khan et al. study no patient required selective embolization or nephrectomy (15). The organs most often injured during PCNL and stone removal are the lungs and pleura, with possible pneumothorax or hydrothorax (29, 30). In our study there was an incidence of 2% to 6% in all the 3 Groups. Bowel perforation can be a serious complication of PCNL puncture. Juan et al. study had a few cases of colon perforation during PCNL (31) which was the same in our study which occurred in a previous open surgery patient on the right side. The patient developed septicemia and died on 5th postoperative day in spite of our broad spectrum antibiotic cover. So we also recommend a pre-operative CT scan so as to study the relationship between the adjoining viscera to the kidney following open surgery as recommended by Margel et al. and Kurtulus et al. (19, 21). Similar to other studies, our study also showed that there are no differences between primary and patients with previous open surgery or PCNL history in terms of stone free rate (SFR) and hospitalization time (8, 12, 20). Overall morbidity ranges from 7.5% to 18% depending on the sample size and the presence of complicated renal stone (32, 33). Overall mortality of PCNL ranges from 0.5% to 1.1% and is generally attributed to severe hemorrhage, urosepsis or pulmonary embolism (23, 32). Accurate reporting of complications is an essential component to critical appraisal and innovation in surgery and specifically in percutaneous nephrolithotomy (PCNL). A standardized complication reporting methodology is necessary to enable appropriate comparisons between institutions, time periods, or innovations in technique (34, 35). The Clavien-Dindo grading system has become widely accepted in urology and has facilitated the study of PCNL complications (36).

## Conclusions

Our single-surgeon experience has proved that PCNL in a patient with a previous history of open nephrolithotomy or PCNL is safe and effective. It can be performed with no fear of higher risk of failure, excessive bleeding, or damage to adjacent organs. Advantages of PCNL in comparison with surgery include cost effectiveness, less complications, less discomfort and increased stone free rate. Prevention rather than treatment is important; thus, we must always make efforts to reduce operation time when performing PCNL.
